# ICH Guideline for Biopharmaceutics Classification System-Based Biowaiver (M9): Toward Harmonization in Latin American Countries

**DOI:** 10.3390/pharmaceutics13030363

**Published:** 2021-03-10

**Authors:** Claudia Miranda, Alexis Aceituno, Mirna Fernández, Gustavo Mendes, Yanina Rodríguez, Verónica Llauró, Miguel Ángel Cabrera-Pérez

**Affiliations:** 1 Centro de Bioactivos Químicos, Unit of Modeling & Experimental Biopharmaceutics, Universidad Central “Marta Abreu” de Las Villas, 54830 Santa Clara, Cuba; cmiranda@uclv.cu; 2 National Drug Agency Department, Institute of Public Health, 7780050 Santiago, Chile; aaceituno@ispch.cl; 3 Faculty of Pharmacy, University of Valparaiso, 2340000 Valparaiso, Chile; 4 Department of Pharmacy, Institute of Pharmacy and Foods, 17100 Havana, Cuba; mirnafc@ifal.uh.cu; 5 General Office of Medicines and Biological Products, Brazilian Health Regulatory Agency, 71205-050 Brasilia, DF, Brazil; Gustavo.Santos@anvisa.gov.br; 6 Laboratorio Nacional de Control, Dirección de Fiscalización y Gestión de Riesgo, Administración Nacional de Medicamentos, Alimentos y Tecnología Médica, C1084AAD Buenos Aires, Argentina; yanina.rodriguez@anmat.gob.ar (Y.R.); veronica.llauro@anmat.gob.ar (V.L.)

**Keywords:** bioequivalence, biowaiver, dissolution, BCS, Latin America, multisource drug

## Abstract

The biopharmaceutical classification system (BCS) is a very important tool to replace the traditional in vivo bioequivalence studies with in vitro dissolution assays during multisource product development. This paper compares the most recent harmonized guideline for biowaivers based on the biopharmaceutics classification system and the BCS regulatory guidelines in Latin America and analyzes the current BCS regulatory requirements and the perspective of the harmonization in the region to develop safe and effective multisource products. Differences and similarities between the official and publicly available BCS guidelines of several Latin American regulatory authorities and the new ICH harmonization guideline were identified and compared. Only Chile, Brazil, Colombia, and Argentina have a more comprehensive BCS guideline, which includes solubility, permeability, and dissolution requirements. Although their regulatory documents have many similarities with the ICH guidelines, there are still major differences in their interpretation and application. This situation is an obstacle to the successful development of safe and effective multisource products in the Latin American region, not only to improve their access to patients at a reasonable cost, but also to develop BCS biowaiver studies that fulfill the quality standards of regulators in developed and emerging markets.

## 1. Introduction

The biopharmaceutical classification system (BCS) is a very important tool to reduce the need for in vivo bioequivalence (BE) studies during the development of new and generic drug products. The BCS is a scientific approach, established by Amidon et al. in 1995, to classify drug substance(s) from immediate release solid orally administered dosage forms, taking into account their aqueous solubility and intestinal permeability properties [1]. The combination of these properties with the dissolution rate of the drug product are considered the most important factors that modulate the rate and amount of the drug absorbed (bioavailability) [2]. Once the drug has been classified, it is possible to establish whether in vitro dissolution tests can replace in vivo bioequivalence studies (biowaiver), eliminating the unnecessary risk of drug exposures to healthy people, accelerating access to the drug product, providing economic relief to industry and governments by decreasing development cost, and maintaining a high public health standard for therapeutic equivalence [3].

Different regulatory authorities and organizations around the world, such as the United States Food and Drug Administration (FDA), the World Health Organization (WHO), and the European Medicines Agency (EMA) have adopted guidelines on the use of BCS-based biowaivers. However, a complete harmonization between them has not been achieved, as significant differences in this regulatory guidance still remain [4,5,6].

Recently, the International Council for the Harmonization of Technical Requirements for Pharmaceuticals for Human Use (ICH M9) has published a consensus guideline for the BCS-based biowaiver with the aim of achieving an understanding on its applicability and the conditions of the waiver, which will apply worldwide [7]. The application of this guideline will reduce in vivo bioequivalence studies in humans, ensuring patients safe and effective medicines.

On the other hand, the application of in vitro bioequivalence requirements by regulatory authorities in Latin America is quite variable [8]. Since 2007, several countries in the region have introduced the BCS-based biowaiver strategies, but the immense majority have not implemented any bioequivalence standards yet [9]. This situation contributes to the prevalence of low-quality and counterfeit medicines in some Latin American and Caribbean countries, posing a challenge for innovative and generic companies seeking to use a BCS biowaiver approach for the global registration of a drug product. In this sense, the purpose of this article is to evaluate the correspondence between the BCS-based biowaiver regulatory guidelines in Latin America and the most recent consensus guideline (ICH M9), and to analyze the perspective of BCS-based biowaiver harmonization in the Latin American region to develop save and effective multisource products.

## 2. Materials and Methods

Official and publicly available guidelines from different Latin America regulatory authorities were considered to analyze information related to the criteria for BCS-based biowaiver application and its role in bioequivalence and therapeutic interchangeability of multisource products (See Table 1). The current regulatory documents were summarized and compared with the most recent harmonized guideline for BCS-based biowaivers proposed by ICH. Recent publications related to BCS-based biowaivers and the impact of harmonization on regulation were also reviewed to identify the most relevant opinions on this process.

Among the main topics to be addressed are those related to the biopharmaceutics classification of the drug substance (solubility and permeability), the drug dissolution rate from the formulation, the excipients and the product.

## 3. Results

### 3.1. Drug Solubility

According to the ICH solubility criteria, a drug substance is considered highly soluble if the highest single therapeutic dose is completely soluble in 250 mL or less of aqueous media in the pH range of 1.2 to 6.8 at 37 °C. 

Table 2 describes a comparison between the ICH solubility requirements for BCS-based biowaivers and those considered by five Latin American regulatory authorities (Chile, Brazil, Cuba, Colombia, and Argentina). As can be seen, the main differences are related to the dose selection to satisfy the high solubility criteria (the highest single therapeutic dose vs. the highest formulation dose strength) and the buffer media required to determine the solubility data. It is also remarkable that several authorities in the Latin American region do not specify, in their regulatory guidance documents, the solubility requirements for BCS biowaivers. This is the first barrier that must be overcome to achieve BCS harmonization in the region.

The generally accepted method for determining the solubility of the drug is the shake-flask method. However, it is not clearly defined in the BCS guidelines of Cuba and Colombia. If justified, other alternative methods would be considered, such as acid or basic titration methods. In all cases, solubility is determined at 37 °C.

The solubility of the drug substance should generally be determined at least at three pH values (pH 1.2, 4.5, and 6.8) as most drugs are weak acids or bases and their solubility depends on both the pKa and the pH of the environment where drugs dissolve. Brazil, Colombia, and Argentina have no pKa requirement for the determination of solubility, while Chile requires additional pHs if the pKa of the drug substances is in the physiological range. When this is the case, the solubility pH dependence is more critical. In fact, for drugs where no pKa is present (non-electrolytes), Chilean regulations do not require a pH-solubility profile. In the case of the ICH guidance, only the pH at which the lowest solubility of the drug is observed is added.

In order to ensure that the solubility at a specific pH value is not influenced by a pH variation during the solubility assay, the pH verification must be performed after the solubility study of the drug substance. This is the process followed by ICH, Brazil, Chile, and Argentina. In the case of Cuba and Colombia, it is not clearly defined.

The ICH regulatory guidance differs partially in the criteria used to determine the solubility class compared to the Latin American regulatory authorities reviewed. The ICH solubility criterion is based on the highest single therapeutic dose, similar to the EMA and WHO guidelines [27,28], while the Latin American regulatory authorities use the highest formulation dose strength, as established by the U.S. FDA guidance [29]. However, if this criterion is not met, but the highest dose strength is soluble in the pH range, a biowaiver may be supported by dose-proportional pharmacokinetics in a range that includes the highest single therapeutic dose. This difference has been supported by the International Consortium for Innovation and Quality in Pharmaceutical Development (IQ), which recommends solubility classification based on the highest formulation dose strength [30].

### 3.2. Drug Permeability

For the ICH permeability criterion, a drug substance is considered highly permeable when the absolute bioavailability is ≥85% or when ≥85% of the administered dose is recovered in urine as the parent drug, or as the sum of the parent drug and Phase 1 and Phase 2 metabolites (oxidative and conjugative metabolites) [7]. This value is required by regulatory authorities in Brazil, Chile, Colombia, and Argentina.

Table 3 shows a comparison between the ICH permeability requirements for BCS-based biowaivers and those set by Latin American regulatory authorities. As can be seen, there are clear differences between the experimental methods approved by some Latin American regulatory authorities for the determination of permeability and the ICH guideline. The ICH, as well as Chile, Colombia, and Argentina, accept as a preferred method the permeability/absorption determined for the approved labelled reference product. In the case of Brazil, since only 21 APIs have been approved for the BCS biowaiver, for these compounds, it is not necessary to demonstrate the permeability values. When the permeability value is not stated for the approved labelled reference product, ICH, Chile, and Argentina justify the complete absorption of the drug with reliable research in humans and through validated permeability assays in Caco-2 cells. Only Colombia considers in vivo intestinal perfusion in humans as an accepted method to achieve complete absorption of the drug. Data from in vivo or in vitro perfusion methods in animal models and validated in vitro Caco-2 permeability are considered supportive methods to demonstrate permeability in Colombia. Only Argentina considers ***in silico*** models as supporting methods for permeability.

Although all of these methods have specific limitations [30], their combined use has proven to be useful in the classification of this property according to the BCS [31]. As in solubility studies, most regulatory authorities in the region did not specify any requirements for determining drug permeability.

### 3.3. Dissolution

Once the high solubility and the high or low permeability of the drug substance have been determined, the dissolution profiles of the test and reference products must demonstrate equally rapid or very rapid dissolution rates under all conditions to be eligible for a BCS-based biowaiver (Class I and III).

As can be seen in Table 4, a comparison between the current dissolution conditions for the ICH regulatory guidance and those set for Latin American regulatory authorities is described. There are clear differences in dissolution testing methods and criteria between the ICH and BCS regulatory guidelines in Latin America. Almost all regulatory authorities in Latin America accept the ICH dissolution criteria for BCS biowaivers (Class I and Class III). Only Brazil considers dissolution requirements for Class I, and countries such as Panama, Venezuela, Costa Rica, and Uruguay did not specify any requirements.

One of the main points of difference between the regulatory authorities is the selection of the dissolution media and buffers. Almost all Latin American regulatory authorities and the ICH accept USP buffers or simulated gastric or intestinal fluids and require dissolution profiles in at least three pH values (1.2, 4.5 and 6.8). The ICH and Colombia also require dissolution profiles at the minimum solubility pH, if this differs from the specified pHs. In the case of Brazil, the use of USP or alternative compendial buffers with the same pH and buffer capacity is recommended.

Surfactants are not accepted by the ICH, Brazil, Chile, Colombia, or Argentina, and must be justified in the cases of Cuba, Costa Rica, and Uruguay. For the rest of the regulatory authorities in Latin American, this is not specified. For the ICH, Brazil, Chile, and Argentina, the use of enzymes is allowed only for gelatin capsules or tablets with a gelatin-based coating (Chilean regulations allow the use of enzymes if crosslinked gelatin is present); it must be justified in the case of Cuba and Uruguay, and it is not considered in the case of Salvador, Ecuador, Panama, Peru, Venezuela, or Costa Rica. Only in the case of Colombia is the use of pepsin at pH 1.2 and pancreatin at pH 6.8 accepted for products containing gelatin.

To determine the similarity of the dissolution profiles of test and reference formulations, the ICH and all Latin American authorities; with the exception of Venezuela, which is not specified; use the similarity factor f2. The ICH, Brazil, Chile, and Ecuador accept the comparison of one batch of the test and reference products; Peru and Argentina recommend two batches; and for the rest of the countries, it is not specified. Regarding the use of dissolution apparatus and rotation speed, the ICH, Brazil, and Chile establish 50 rpm for the USP II apparatus (paddle); however, when a coning effect appears at this speed, some alternative approaches are justified. Cuba, Colombia, Ecuador, Argentina, Costa Rica, and Uruguay allow a faster paddle speed of up to 75 rpm to reduce coning. The rotation speed of the basket is usually 100 rpm. In the case of Salvador, Panama, Peru, and Venezuela, no information is declared.

In the case of the ICH, the sampling time points are not reported, while in the rest there are slight differences. Cuba, Costa Rica, and Uruguay recommend 10, 15, 20, 30, 45, and 60 min, Chile and Colombia recommend an additional sampling time point of 5 min, while Salvador, Panama, and Peru do not specify it. Finally, the ICH, Brazil, Cuba, Colombia, and Argentina require that the bath temperature is 37 ± 1 °C, Chile and Costa Rica state that they require 37 ± 0.5 °C, while the remainder do not specify a temperature.

### 3.4. Excipients

Table 5 shows a comparison between the ICH excipient requirements for BCS-based biowaivers and those established by regulatory authorities in Latin America. This is a very important point because differences in the excipients between the test and reference products that affect the solubility, dissolution, and in vivo absorption of the drug substance (e.g., mannitol, sorbitol, surfactants, etc.) must be identified. In this case, the ICH proposes a more careful evaluation of critical excipients. For Class I, the critical excipients should be qualitatively the same and quantitatively similar (±10% of the amount of excipient in the reference product); for Class III, the range is stricter, with ±10% of the amount of excipient in the reference product and the cumulative difference for these excipients required to be within ±10%. On the other hand, some regulatory authorities in Latin America do not specify the quantity of excipients (Cuba, Panama, Venezuela, Costa Rica, and Uruguay), while others limit their use only to excipients that do not affect bioavailability (Salvador, Ecuador, Peru, and Argentina).

### 3.5. Product

The ICH and almost all regulatory authorities in Latin America, with the exception of Panama, agree that BCS-based biowaivers are limited to the immediate release (IR) oral dosage form with systemic action.

Regarding the choice of API for BCS-based biowaivers, only the ICH, Brazil, Chile, and Argentina are declared eligible when the active part of the drug substance in the test and reference products is identical (See Table 5). However, BCS-based biowaivers for different esters, ethers, isomers, mixtures of isomers, complexes or derivatives of the drug would not be accepted by any participant, as these differences could result in differences in bioavailability that would not be detectable using the BCS-based biowaiver criteria. For the rest of the regulatory authorities in Latin America, this information is not declared.

## 4. Discussion

The BCS is a scientific tool that was created to identify possible biowaivers to replace unnecessary in vivo bioequivalence studies [1]. Its wide international use has led to the recent publication by the ICH of a consensus guideline for the worldwide applicability of BCS. In the Latin American region, only a few countries apply the BCS approach and not all of them consider this framework to the biowaiver API belonging to BCS I (high solubility and high permeability) and III (high solubility and low permeability) classes [9]. Compounds classified as BCS Class II (low solubility and high permeability) and IV (low solubility and low permeability) are not considered for the biowaiver by the main regulatory agencies and organizations.

Some of the Latin American regulatory agencies are known to have implemented BCS biowaivers following the requirements of the US-FDA or WHO guidelines [4]. A clear comparison between both jurisdictions with the Latin American BCS guidelines has been previously published and several differences have been found, such as the dose used to demonstrate high aqueous solubility, the eligible methods for permeability determination, the agitation rate, and the volume used for dissolution tests, among others [9]. In this sense, this article provides a point of view on the perspective of harmonization of BCS-based biowaivers in the Latin American region through a comparison with the most recent ICH M9 guideline. 

The results reveal that only Chile, Brazil, Colombia, and Argentina have a more comprehensive BCS guideline, including solubility, permeability and dissolution requirements. Although there are many similarities between them and the ICH M9 guideline, there are still big differences in the interpretation and implementation. At this point, it must be pointed out that Latin America does not have a regional regulatory authority and every country is an independent jurisdiction, urging the need to create a regional regulatory body as in Europe.

One of the most important aspects of the harmonization of BCS-based biowaivers is the selection of the dose for solubility classification. Both criteria—the highest single dose and the highest dose strength—are questionable. Firstly, the selection of one or the other dose definition can affect the solubility classification of an API, which leads in some cases to an API having different BCS classes depending on the dose considered (e.g., metoclopramide, verapamil), discarding from a possible BCS-based biowaiver [6,32]. On the other hand, it is known that in bioequivalence studies, the dose used is the highest of the product (dose strength), so a harmonization of the dissolution tests for the biowaiver would justify the use of this dose. Although the “highest single dose” is the maximum dose that the patient should use for a pharmaceutical product, no experimental data are described in the literature that demonstrate that the highest single dose is more discriminatory than the highest dose strength of the product. Furthermore, from a practical point of view, the use of multiple dosage form units in a single dissolution vessel can produce changes in the vessel hydrodynamics and pH of the medium, which would increase the variability of the results [6].

Another relevant factor to consider is the variability of permeability methods that will be accepted to support a permeability classification. Although the mass balance study is the unequivocally accepted method for determining permeability, this type of study presents several problems. Among them are those related to the formulation used, the limitation of oral studies to miss the parent compound absorbed and secreted into feces via bile, and the lower recovery of well-absorbed compounds with long plasma radioactivity half-lives [33]. Recently, Bransford et al. have recommended the use of an all-evidence approach to harmonize permeability criteria, given priority to human data over animal or in vitro data [30]. 

In relation to dissolution studies, there are slight differences between the ICH M9 and the BCS-based biowaivers guidelines in Latin America. To obtain a further harmonization, it is necessary to develop comparative in vitro dissolution experiments using compendial apparatus (USP I and USP II), validated analytical methods and standard experimental conditions (rotation speed: paddle 50 rpm and basket 100 rpm; pharmacopoeia buffers, at least pH 1.2, 4.5, and 6.8; 900 mL or less of media at 37 °C; at least 12 units of test and reference product for each dissolution profile; not using organic solvents or surfactants and enzymes may be acceptable). However, none of the BCS regulatory guidelines mention the selection of a drug comparison product for the dissolution study. The need for a harmonized list of comparators has been demonstrated by several authors [6]. International harmonization of comparators may be necessary to ensure equal performance of generic drug products in any region where they are applied. The use of innovator products from well-regulated markets have been suggested as the best option for BCS-based biowaivers [34]. Although it is not in the scope of this manuscript, the inequalities of the criteria to accept dose proportionality biowaiver studies also needs to be explored and addressed.

The ICH M9 and some BCS guidelines from Latin America contain general requirements on excipients that apply to all drugs within a particular BCS class. The variability in the amount of excipient allowed between test and reference formulations is different between BCS classes I and III, and also between the regulatory authorities in Latin America. At the same time, a limited number of critical excipients have been identified that affect compounds belonging to BCS classes I and III [35]. Although most of the excipients used in solid oral IR dosage formulations do not have a relevant influence on the in vivo absorption of a drug, a detailed analysis of critical excipients should be developed, in particular for BCS Class III compounds in order to avoid rejections of changes to the formulation that will bioequivalent in vivo [35].

The ICH M9 provides recommendations to support biowaivers of in vivo bioequivalence studies of drug products based on BCS. This guideline considered all of the relevant aspects of other major guidance documents (FDA, EMA, WHO, etc.) and included new topics to give it more flexibility and international applicability [6]. However, as it can be seen in the present study, of the few Latin American countries that have implemented the BCS-based biowaiver to demonstrate the interchangeability of multisource drugs [9], only Chile, Brazil, Colombia, and Argentina bring information on drug solubility, permeability and dissolution into their BCS guidelines, but their information is slightly different among them and also compared to the ICH M9. This result highlights the challenges that the Latin American region has to overcome to improve the development of the BCS-based biowaiver and its application in the development of safe and effective medicines at a reasonable cost.

## 5. Conclusions

The ICH M9 is a step forward in establishing international harmonization for the BCS-based biowaiver of multisource products. Its global application will reduce the costly and time-consuming in vivo bioequivalence studies in humans and provide good quality multisource products at lower cost for the benefit of patients worldwide. Although few Latin American countries have applied the BCS-based biowaiver to demonstrate the interchangeability of multisource drugs, only Chile, Brazil, Colombia, and Argentina show good convergence with the ICH guideline. This situation is an obstacle for the good development of safe and effective multisource products in the Latin American region, not only to improve their access to patients at a reasonable cost, but also to develop BCS biowaiver studies that fulfill the quality standards of regulators in developed and emerging markets.

## Figures and Tables

**Table 1:** Official and publicly available Biopharmaceutical Classification System (BCS) guidelines from the International Council for the Harmonization of Technical Requirements for Pharmaceuticals for Human Use (ICH) and Latin American regulatory authorities.

Regulatory Authority	Country	BCS Guideline (s) Referenced
ICH		The International Council for Harmonisation of Technical Requirements for Pharmaceuticals for Human Use. ICH Harmonised Guideline M9: biopharmaceutics classification system-based biowaivers [7].
ANVISA	Brazil	The first guideline that included BCS Biowaivers in Brazil was Resolution n. 37, of 3 August 2011 [10].
ISP	Chile	Since 2007, a guidance entitled G-BIOF02 “Biowaiver of Bioavailability/Bioequivalence Studies to establish Therapeutic Equivalence of Oral Solid Dosage Forms” has been in use to guide sponsors for BCS based biowaivers. Latest version 2018 [11].
CECMED	Cuba	Regulation 18-07: requirements for bioavailability and bioequivalence studies (2007) [12]. Regulation No. 48/2007: requirements for applying and/or designing a dissolution test in capsules and tablets of immediate release (2007) [13].
INVIMA	Colombia	Resolution 1124/2016 Guide containing criteria and requirements for the study of bioavailability and bioequivalence of drugs (2016) [14].
DNM	Salvador	Salvadorian technical regulations: (RTS 11.02.01:16), pharmaceutical products; medicines for human use. Bioequivalence and interchangeability (2016) [15].
ARCSA	Ecuador	Health registration replacement regulation for medicines in general (Agreement No. 00000586)/Reform 2016 [16].
DNFD	Panama	Amendment of executive decree No. 6 of 2005 on therapeutic equivalence and interchangeability (2010) [17]. Law 1/2001, about medicines and other products for human health (2001) [18].
DIGEMID	Peru	Supreme decree No 024-2018-SA: regulations governing the interchangeability of medicines (2018) [19].
INHRR	Venezuela	Resolution No 212, by which the Venezuelan norms of bioavailability and bioequivalence of pharmaceutical products are dictated (2006) [20].
ANMAT	Argentina	Disposition 758/2009: biowaiver criteria for bioequivalence studies for immediate release oral solid drugs [21]. Disposition 5068/2019: guidance for applying for a biowaiver of active pharmaceutical ingredients with bioequivalence requirement. This is the last version of the Disposition 6766/2016 [22].
MINSA	Costa Rica	Technical guide for the presentation and evaluation of comparative dissolution profile studies (2009) [23]. Decree 32470-S/ 2005: regulations for the health registration of medicines that require the demonstration of therapeutics equivalence [24].
MSP	Uruguay	Decree No. 87/016: amendment of decree No. 12/007 on the interchangeability of medicines (2016) [25]. Decree 12/007: interchangeability of medicines (2007) [26].

ANVISA: Agencia Nacional de Vigilancia Sanitaria; ISP: Instituto de Salud Pública; CECMED: Centro para el control estatal de medicamentos y dispositivos médicos; INVIMA: Instituto Nacional de Vigilancia de Medicamentos y Alimentos; ARCSA: Agencia Nacional de Regulación, Control y Vigilancia Sanitaria; DIGEMID: Dirección General de Medicamentos Insumos y Drogas; ANMAT: Administración Nacional de Medicamentos, Alimentos y Tecnología; DNM: Dirección Nacional de Medicamentos; INHRR: Instituto Nacional de Higiene Rafael Rangel; MINSA: Ministerio de Salud; MSP: Ministerio de Salud Pública; DNFD: Dirección Nacional de Farmacias y Drogas

**Table 2:** Comparison of solubility requirements for BCS-based biowaivers between ICH and Latin American regulatory authorities.

Parameter	Regulatory Authority						
	ICH M9	ANVISA	ISP	CECMED	INVIMA	ANMAT	Comments
Method	Shake-flask or other justified method	Y	Y	ND	ND	Y	The solubility requirements are not specified in other regulatory authorities such as: DNM, ARCSA, DNFD, DIGEMID, INHRR, MINSA and MSP
Dose (unit studied)	Highest single therapeutic dose or,	N	N	N	N	N	
	Highest dose strength supported by dose-proportional for biowaiver	Y	Y	Y	Y	Y	
Volume	Soluble in 250 mL or less of aqueous media in the pH range	Y	Y	ND	Y	Y	
Replicates	A minimum of three replicates at each solubility condition/pH	Y	Y	Y	Y	Y	
pH	At least three pH values: 1.2, 4.5, 6.8; and	Y	Y, and pH = pKa and pH = pKa ± 1	ND	Y	Y	
	at the pH at which the lowest solubility of the drug is observed	N	N	ND	N	N	
Timing of pH measure	Before and after addition of the drug	Y	Y	ND	ND	Y	
Temperature	37 ± 1 °C	Y	37 ± 0.5 °C	Y	Y	Y	
Media	Buffer solutions at pH 1.2, 4.5, 6.8, and pKa or pI if within pH 1.2−6.8	Buffers from Brazilian Pharmacopoeia or other official compendia recognized by ANVISA	Y	ND	Y	Pharmacopoeia buffers between pH 1.2 and 6.8	
Origin of data	Sponsor	Y	Authorized BCS centers	ND	ND	Y	

ANVISA: Agencia Nacional de Vigilancia Sanitaria (Brazil); ISP: Instituto de Salud Pública (Chile); CECMED: Centro para el control estatal de medicamentos y dispositivos médicos (Cuba); INVIMA: Instituto Nacional de Vigilancia de Medicamentos y Alimentos (Colombia); ARCSA: Agencia Nacional de Regulación, Control y Vigilancia Sanitaria (Ecuador); DIGEMID: Dirección General de Medicamentos Insumos y Drogas (Perú); ANMAT: Administración Nacional de Medicamentos, Alimentos y Tecnología (Argentina); DNM: Dirección Nacional de Medicamentos (El Salvador); INHRR: Instituto Nacional de Higiene Rafael Rangel (Venezuela); MINSA: Ministerio de Salud (Costa Rica); MSP: Ministerio de Salud Pública (Uruguay); DNFD: Dirección Nacional de Farmacias y Drogas (Panamá). N: No (Disagree to ICH), Y: Yes (Agree to ICH), ND: Not defined.

**Table 3:** Comparison of permeability requirements for BCS-based biowaivers between ICH and Latin American regulatory authorities.

Parameter	Regulatory Authority					
	ICH M9	ANVISA	ISP	INVIMA	ANMAT	Comments
High permeability	≥85% of drug substance absorption	Y	Y	Y	Y	The permeability requirements are not specified in other regulatory authorities such as: CECMED, DNM, ARCSA, DNFD, DIGEMID, INHRR, MINSA and MSP
Preferred Method	Absolute bioavailability or mass balance in humans.	N	Y	Y	Y	
Accepted Method	Human in vivo data from published literature.	N	Y	In vivo intestinal perfusion in humans	Y	
	Validated in vitro Caco-2 permeability assays.	N	Y	N	Y	
Supportive Method	ND	N	ND	In vivo or in vitro perfusion methods in animal models. In vitro Caco-2 assays	In silico methods	
Stability of drug in the GIT	Demonstrated for in vitro Caco-2 permeability and for mass balance studies.	N	Y	ND	GTI fluids from animal and/or simulated GTI Fluids USP or GTI human fluids	

ANVISA: Agencia Nacional de Vigilancia Sanitaria (Brazil); ISP: Instituto de Salud Pública (Chile); CECMED: Centro para el control estatal de medicamentos y dispositivos médicos (Cuba); INVIMA: Instituto Nacional de Vigilancia de Medicamentos y Alimentos (Colombia); ARCSA: Agencia Nacional de Regulación, Control y Vigilancia Sanitaria (Ecuador); DIGEMID: Dirección General de Medicamentos Insumos y Drogas (Perú); ANMAT: Administración Nacional de Medicamentos, Alimentos y Tecnología (Argentina); DNM: Dirección Nacional de Medicamentos (El Salvador); INHRR: Instituto Nacional de Higiene Rafael Rangel (Venezuela); MINSA: Ministerio de Salud (Costa Rica); MSP: Ministerio de Salud Pública (Uruguay); DNFD: Dirección Nacional de Farmacias y Drogas (Panamá). GTI: gastrointestinal; N: No (Disagree to ICH), Y: Yes (Agree to ICH), ND: Not defined.

**Table 4:** Comparison of dissolution requirements for BCS-based biowaivers between ICH and Latin American regulatory authorities.

Parameter	ICH M9	Other Criteria	Agree with ICH	Disagree with ICH	Not Defined
Profile	Class I: very rapid ≥85% in ≤15 min., or rapid ≥85% in ≤30 min.		ANVISA, ISP, CECMED, INVIMA, DNM, ARCSA, DIGEMID, ANMAT		DNFD, INHRR, MINSA, MSP
	Class III: very rapid ≥85% in ≤15 min.		ISP, CECMED, INVIMA, DNM, ARCSA, DIGEMID, ANMAT	ANVISA	DNFD, INHRR, MINSA, MSP
Apparatus and rotation	USP I (basket): 100 rpm		ANVISA, ISP, INVIMA, DNM, ARCSA, ANMAT, MINSA, MSP		DNM, DNFD, DIGEMID, INHRR
		50–100 rpm	CECMED		
	USP II (paddle): 50 rpm				DNM, DNFD, DIGEMID, INHRR
		75 rpm	INVIMA, ARCSA, ANMAT, MINSA, MSP		
		50–75 rpm	CECMED		
Medium volume	≤900 mL		ANVISA, ISP, CECMED, INVIMA, ANMAT, MINSA, MSP		DNM, DNFD, DIGEMID, INHRR
Temperature	37 ± 1 °C		ANVISA, CECMED, INVIMA, ANMAT		DNM, ARCSA, DNFD, DIGEMID, INHRR
		37 ± 0.5 °C	ISP, MINSA		
		37°C	MSP		
Type of medium	Three buffers: pH 1.2, 4.5, 6.8		ANVISA, ISP, CECMED, INVIMA, DNM, ARCSA, MSP		DNFD, DIGEMID, INHRR
		simulated gastric fluid (pH 1.2), simulated intestinal fluid (pH 6.8)	ANVISA, CECMED, MINSA		
		Other buffers may be acceptable if justified	ANVISA *, ISP, ANMAT		
		pH range should include the pKa region	DNM		
	Also, at the pH of lowest solubility (if different from the buffers above)			ANVISA, ISP, CECMED, INVIMA, DNM, ARCSA, DNFD, DIGEMID, INHRR, ANMAT, MINSA, MSP	
Use of enzyme	Only for gelatin capsules or tablets with gelatin coatings		ANVISA, ISP, ANMAT		DNM, ARCSA, DNFD, DIGEMID, INHRR, MINSA
		Pepsin should be justified	CECMED, MSP		
		at pH 1.2 and pancreatin at pH 6.8 in products containing gelatin	INVIMA		
Use of surfactant	No		ANVISA, ISP, INVIMA, ANMAT		DNM, ARCSA, DNFD, DIGEMID, INHRR
		It should be justified	CECMED, MINSA, MSP		
Comparative test	Similarity factor f2 ≥ 50		ANVISA, ISP, CECMED, INVIMA, DNM, ARCSA, DNFD, DIGEMID, ANMAT, MINSA, MSP		INHRR
Sampling time	Not declared		ANVISA		DNM, DNFD, DIGEMID, INHRR
		10, 15, 20, 30, 45 min.	ANMAT		
		10, 15, 20, 30, 45, 60 min.	CECMED, MINSA, MSP		
		5, 10, 15, 20, 30, 45, 60 min.	INVIMA		
		5, 10, 15, 20, 25, 30, 45, 60 min.	ISP		
		Four sample times (not t = 0). One point after 85% dissolved	ARCSA		
Number of batches	One		ANVISA, ISP		CECMED, INVIMA, DNM, DNFD, INHRR, MINSA, MSP
		Two	DIGEMID, ANMAT		
Unit tested	At least 12 units of reference and test products		ANVISA; ISP; CECMED, INVIMA, DNM, DNFD, DIGEMID, INHRR, ANMAT, MSP		ARCSA, MINSA
Fixed dose combination	All APIs must comply		DNM, DNFD, DIGEMID, ANMAT, MSP	ISP	CECMED, INVIMA, ARCSA, INHRR, MINSA
		If one API is not a BCS-based biowaiver, in vivo BE is needed	ANVISA		
Biowaiver for other strengths	Yes (under certain conditions)		ANVISA, ISP, INVIMA, DNM, DIGEMID, ANMAT, MSP		CECMED, ARCSA, DNFD, INHRR, MINSA

ANVISA: Agencia Nacional de Vigilancia Sanitaria (Brazil); ISP: Instituto de Salud Pública (Chile); CECMED: Centro para el control estatal de medicamentos y dispositivos médicos (Cuba); INVIMA: Instituto Nacional de Vigilancia de Medicamentos y Alimentos (Colombia); ARCSA: Agencia Nacional de Regulación, Control y Vigilancia Sanitaria (Ecuador); DIGEMID: Dirección General de Medicamentos Insumos y Drogas (Perú); ANMAT: Administración Nacional de Medicamentos, Alimentos y Tecnología (Argentina); DNM: Dirección Nacional de Medicamentos (El Salvador); INHRR: Instituto Nacional de Higiene Rafael Rangel (Venezuela); MINSA: Ministerio de Salud (Costa Rica); MSP: Ministerio de Salud Pública (Uruguay); DNFD: Dirección Nacional de Farmacias y Drogas (Panamá). N: No (Disagree to ICH), Y: Yes (Agree to ICH), ND: Not defined. * Dissolution media described in the Brazilian Pharmacopoeia or other official compendia recognized by ANVISA.

**Table 5:** Comparison of excipient, dosage form and API requirements between ICH and Latin American regulatory authorities.

Regulatory Authority	Parameters		
	Dosage Form	Acceptable Excipients (for classes)	API
ICH	IR oral dosage form with systemic action, and the drug product is the same dosage form and strength as the reference product. For FDC products when all drug substances contained in the combination drug product are class I and/or III	Class I: qualitative and quantitative differences in excipients are permitted, except for excipients that may affect absorption, which should be qualitatively the same and quantitatively similar, i.e., within ±10% of the amount of excipient in the reference product. Class III: all of the excipients should be qualitatively the same and quantitatively similar (except for film coating or capsule shell excipients). Excipients that may affect absorption should be qualitatively the same and quantitatively similar, i.e., within ±10% of the amount of excipient in the reference product, and the cumulative difference for these excipients should be within ±10%	Eligible when the drug substance(s) in test and reference products are identical; eligible if test and reference products contain different salts provided that both belong to BCS Class I; not eligible when the test product contains a different ester, ether, isomer, mixture of isomers, complex or derivative of a drug substance from that of the reference product
ANVISA	IR oral dosages only	It is recommended that the test formulation employ the same excipients as in the formulation of the reference drug. The applicant must provide information about the function of each excipient, as well as justification of the amount used. If excipients that are proven to affect the bioavailability of drugs are used, the test drug should contain, with respect to these excipients, qualitatively the same as the reference medicine and in an amount compatible with the intended function in the pharmaceutical form.	Eligible when the drug substance(s) in test and reference products are identical; not eligible when the test product contains a different salt, ester, ether, isomer, mixture of isomers, complex or derivative of a drug substance from that of the reference product
ISP	Idem, except for FDCs where there is no consensus as how to apply BCS biowaiver to these types of formulations. Guidance also allows in vitro comparison for controlled released products in case of dose strength proportionality biowaiver	Class I: There is flexibility regarding Q1 and Q2 for traditional excipients. New or excessive quantities of one excipient must be justified. It is advisable that for known excipients affecting permeability Q2 be the same as in the reference product. Class III: Formulations should be qualitatively the same and quantitatively similar (there is a limit permitted for differences in all excipients, unless experimental data is provided showing no effect on permeability). Effects of excipients and limits are also applicable to aqueous formulations.	Y
CECMED	IR oral dosage with systemic action	ND	ND
INVIMA	IR oral dosage with systemic action	Class I: Flexibility for excipients used, except for critical excipients. Class III: All excipients must be qualitatively the same and quantitatively similar to those of the reference product (limits established by WHO)	ND
DNM	Multisource drugs with oral administration and conventional release. Different concentration of the same product provided that a bioequivalence has been demonstrated for one of them	Excipients that do not affect the bioavailability.	provides an API list that has been classified based on BCS and sanitary risk criterion
ARCSA	IR oral dosage form that meets the BSC requirement	Excipients that do not affect the bioavailability.	ND
DNFD	ND	ND	ND
DIGEMID	IR oral dosage	Excipients that do not affect the absorption of drugs.	ND
INHRR	APIs in suspension and oral administration with conventional release	ND	ND
ANMAT	IR oral dosage form with systemic action, and the drug product is the same dosage form and strength as the reference product. For FDC products when all drug substances contained in the combination are class I and/or III	For class I drugs, there is flexibility except for excipients that may affect absorption. For class III drugs, formulations should be qualitatively the same and quantitatively similar (there is a limit permitted for differences in all excipients)	Eligible when the active part of the drug substance(s) in test and reference products are identical. It may contain different salts or different ester, or complex of a drug substance from that of the reference
MINSA	IR oral dosage form	ND	ND
MSP	IR oral dosage form	ND	ND

ANVISA: Agencia Nacional de Vigilancia Sanitaria (Brazil); ISP: Instituto de Salud Pública (Chile); CECMED: Centro para el control estatal de medicamentos y dispositivos médicos (Cuba); INVIMA: Instituto Nacional de Vigilancia de Medicamentos y Alimentos (Colombia); ARCSA: Agencia Nacional de Regulación, Control y Vigilancia Sanitaria (Ecuador); DIGEMID: Dirección General de Medicamentos Insumos y Drogas (Perú); ANMAT: Administración Nacional de Medicamentos, Alimentos y Tecnología (Argentina); DNM: Dirección Nacional de Medicamentos (El Salvador); INHRR: Instituto Nacional de Higiene Rafael Rangel (Venezuela); MINSA: Ministerio de Salud (Costa Rica); MSP: Ministerio de Salud Pública (Uruguay); DNFD: Dirección Nacional de Farmacias y Drogas (Panamá). N: No (Disagree to ICH), Y: Yes (Agree to ICH), ND: Not defined; FDC: fixed dose combination; IR: Immediately release.

## Data Availability

Not applicable.
